# Using Immersive Simulation to Engage Pediatric Residents in Difficult Conversations and the Disclosure of Patient Safety Events

**DOI:** 10.7759/cureus.3095

**Published:** 2018-08-03

**Authors:** Dana A Schinasi, Irini N Kolaitis, Frances M Nadel, Yuemi An-Grogan, Rebekah Burns, Leah Berman, Annie M Quinn, Kathy N Shaw

**Affiliations:** 1 Pediatrics, Ann & Robert H Lurie Children's Hospital of Chicago/Northwestern University Feinberg School of Medicine, Chicago, USA; 2 Hospital Based Medicine, Northwestern University Feinberg School of Medicine/Ann & Robert H. Lurie Children's Hospital of Chicago, Chicago, USA; 3 Pediatric Emergency Medicine, The Children's Hospital of Philadelphia, Philadelphia, USA; 4 Pediatric Emergency Medicine, Ann & Robert H Lurie Children's Hospital of Chicago/Northwestern University Feinberg School of Medicine, Chicago, USA; 5 Pediatrics, Seattle Children's Hospital/University of Washington School of Medicine, Seattle, USA; 6 Pediatric Emergency Medicine, Ann & Robert H Lurie Children's Hospital of Chicago, Chicago, USA; 7 Pediatric Emergency Medicine, Perelman School of Medicine/university of Pennsylvania, Philadelphia, USA

**Keywords:** disclosure, difficult conversations, simulation, patient safety events

## Abstract

Background

Full disclosure of patient safety events (PSE) is desired by patients and their families, is required by the Joint Commission and many state laws, and is vital to improving patient outcomes. A key barrier to consistent disclosure of patient safety events is a self-reported lack of proper training. Physicians must be trained to recognize when a PSE has occurred and effectively carry out disclosure, all while caring for a patient who is actively experiencing the consequences of an unintended outcome. Immersive simulation provides the opportunity to practice this complex skill.

Objective

To develop and evaluate a simulation-based workshop for pediatric residents on the disclosure of patient safety events.

Methods

A workshop in PSE disclosure was developed according to literature review, expert consultation, and feedback from hospital administration. The three-hour workshop included a simulated PSE with a subsequent standardized debriefing, interactive didactic session, and additional simulation-based hands-on practice in disclosure. Participants completed an anonymous survey at one-week and three-months post workshop, assessing workshop satisfaction, subsequent clinical experience, and perceived change to their practice.

Results

During the one-year study period, 27/31 (87.0%) second year residents completed the workshop. At the one-week follow-up, all study participants reported increased confidence and preparedness in their ability to lead the initial disclosure conversation. All study participants felt that the simulated scenarios were realistic and relevant to their current clinical duties and 33.3% (n=9) stated that they would like to repeat this workshop prior to completion of their training. At the three-month follow-up, 29.6% (N=8) of study participants reported involvement in the disclosure of a patient safety event since the workshop with all eight reporting feeling adequately prepared by the workshop for this experience. Study participants indicated that post training they were more likely to engage the attending physician, risk management and patient relations in the disclosure conversation (p <=0.05). The estimated cost of this simulation training for 27 residents was $6,993, not accounting for the 39 hours per clinician facilitator.

Conclusions

Immersive simulation is uniquely suited for teaching difficult conversation skills that are encountered during acute care, including the disclosure of patient safety events. While hands-on practice is critical, faculty and simulation resources required for continued implementation may not be sustainable long-term. Future training curricula should leverage creative and innovative adult-learning techniques to reach a wide range of members of the care team with less resource utilization.

## Introduction

Since 2001, the Joint Commission on Accreditation of Healthcare Organizations (JCAHO) has required hospitals to disclose all unexpected outcomes, a practice that has been written into many states’ laws [1]. Physicians have an ethical duty to disclose patient safety events (PSE) to patients and families [2,3], providing the patient and family with an explanation of what happened and the assurance that steps are being taken to prevent a similar occurrence in the future. Moreover, full disclosure of PSE is desired by patients and their families and is vital to improving overall patient satisfaction [4-6]. When a safety event occurs in clinical practice, pediatricians support complete transparency [7]. Yet surveys suggest that few PSE are disclosed to patients, and disclosure conversations often fail to meet patient expectations. Failure to disclose PSE promptly and properly can disrupt patient-clinician relationships and may increase the chance of malpractice lawsuits [8]. Good communication is essential to providers and patients and their families alike, particularly in the face of unanticipated events.

There are many potential barriers to the consistent and transparent disclosure of PSE by physicians, including fear of malpractice litigation, concern for implications to their professional reputation, and lack of proper training [9-11]. Physicians must be adequately trained to recognize when a PSE has occurred and to effectively carry out disclosure consistent with institutional policies [12]. This notion is further supported in a study of pediatric attending and resident physicians, which concluded that training programs should include formal instruction on disclosure and offer the opportunity to both practice these skills and receive feedback [7]. Many institutional-specific policies exist to guide the process of disclosure. According to the Ann & Robert H. Lurie Children’s Hospital of Chicago Administrative Policy on Communication of Patient Outcomes and Unanticipated Events, “the Attending Physician or his/her designee” is charged with the task of disclosure [13]. Our institution currently addresses this training need with an online module, which includes vignettes with examples of PSE but does not provide instruction on how to initiate and conduct the disclosure in its entirety [14].

Simulation is a powerful tool in medical education because it offers trainees an opportunity to explore new skills and make mistakes in a safe learning environment and receive corrective feedback as a guide for future practice [15]. Another main benefit of simulation is that structured debriefing allows trainees an opportunity for reflection leading to formation of ideas to improve their performance in future simulations and/or clinical practice. In addition, facilitators have a unique opportunity to hone in on areas for improvement in the participants’ communication style and teamwork. A simulation-based workshop in disclosure of PSE has the potential to complement the current practice of didactic education, providing physicians with the opportunity for hands-on practice of this complex skill. Lastly, a disclosure workshop for resident physicians would address several of the core competencies mandated in the Graduate Medical Education guidelines [16], including communication skills and professionalism.

The aims of this study were as follows: (1) to describe the efforts to design and implement a simulation-based workshop for pediatrics residents; (2) to investigate its impact by collecting data on participant experience, impressions, and feedback; and (3) to assess feasibility of program sustainability.

## Materials and methods

Workshop development

A workshop on the disclosure of patient safety events was developed by the authors according to Kern’s Six Steps of Curriculum development [17]. As previously mentioned, it is known that PSE relating to human factors and systematic biases prevail throughout our health care systems, despite efforts to minimize them. Current practices and curricula at our institution lacked dedicated and targeted instruction regarding disclosure, especially for residents. Background and support for the need of a disclosure curriculum included a literature review, incorporation of previous workshop experience, consultation with experts in the field, and feedback from hospital administration. The objectives of the workshop were as follows: (1) to recognize when a PSE has occurred, (2) develop a clear understanding of legal considerations and institutional policies, (3) understand the value of disclosure, (4) review best practices for disclosure, and (5) gain practical experience in disclosure. We then used a blended-learning educational strategy to disseminate our curriculum, which consisted of didactics, high-fidelity simulation cases, and interactive small-group discussions. The program was implemented after residency leadership and institutional support was obtained. Barriers to implementation included both faculty and learner time, and simulation center availability. Post-session questionnaires and program evaluations were obtained.

**Figure 1:**
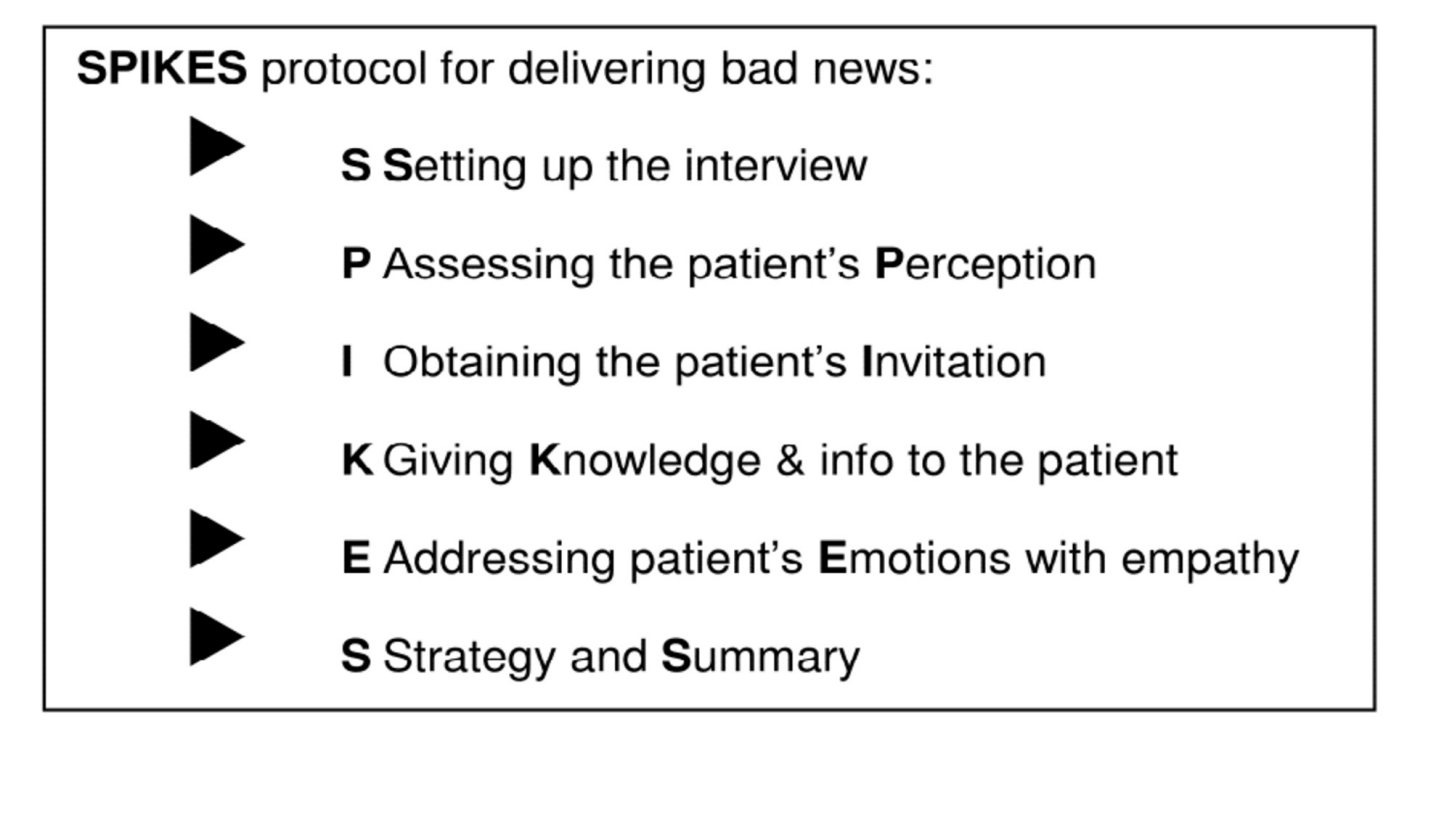
SPIKES protocol for delivering bad news [17]

Based on a comprehensive review of the literature, the SPIKES protocol was chosen as a framework for the disclosure conversation. The SPIKES protocol was developed for oncology patients and used to help with disclosing unfavorable information—"breaking bad news"—to cancer patients about their illness [18], and as such is well-suited for the difficult PSE disclosure conversation. The SPIKES protocol is simple and straightforward and consists of six steps (Figure 1). The goal is to enable the clinician to fulfill the four most important objectives while disclosing difficult news, which include (1) gathering information from the patient; (2) transmitting the medical information; (3) providing support to the patient; (4) eliciting the patient's collaboration in developing a strategy or treatment plan for the future.

**Figure 2:**
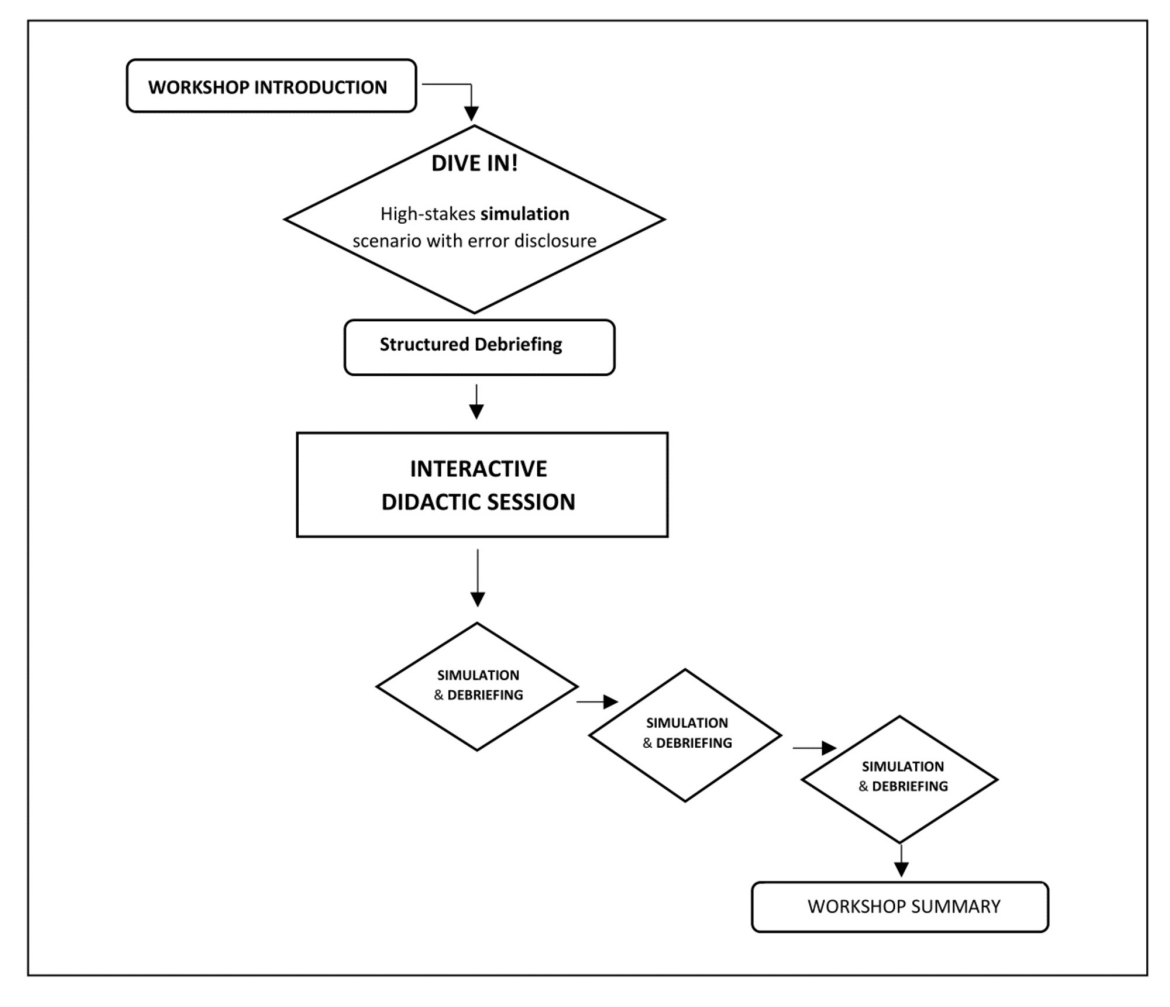
Workshop structure and flow

Our three-hour disclosure workshop begins with a simulated PSE as part of a critically ill patient scenario, requiring the participant to provide medical care while simultaneously dealing with complex emotions experienced by the simulated patient’s parent and bedside nurse. Participants then engage in standardized debriefing and an interactive didactic session. The workshop concludes with three additional simulation-based scenarios, each followed by a structured debriefing. The workshop outline and flow are detailed in Figure 2. The workshop was designed to accommodate two-to-three participants per session and requires one lead facilitator, two confederates (nurse, parent), and one behind-the-scenes simulation technician to provide a comprehensive experience. A total of 11 second-year pediatrics residents participated in five pilot sessions during the 2013-2014 academic year at Ann & Robert H. Lurie Children’s Hospital of Chicago. Feedback from these pilot sessions helped to further refine the workshop prior to the start of this study.

Participant recruitment

During the 2014-2015 academic year, all second-year residents in the pediatrics residency training program at Ann & Robert H. Lurie Children’s Hospital of Chicago/Northwestern Feinberg School of Medicine were approached to participate in this study. This workshop was included as part of the Community Medicine and Advocacy rotation during the second year of pediatrics residency. Prior to the start of the workshop, all study participants were able to review and complete an informed consent document that outlined the study goals and the workshop objectives and included resources to contact if any of the workshop content was distressing to participants. Residents were informed that participation in this study was voluntary and would not impact their training education or evaluation. All study participants were able to opt out from enrolling in the study but could still participate in the educational experience. Study participants who were unable to attend their scheduled session were provided with dates for make-up sessions. This study was approved by the Institutional Review Board at Ann & Robert H. Lurie Children’s Hospital of Chicago.

Study procedures

One week following workshop completion, participants received an internet-based questionnaire assessing their satisfaction with the overall workshop, simulation components, as well as how the workshop has or will change their practice. A similar electronic follow-up questionnaire was sent to participants three months following completion of the workshop in order to assess practice patterns and residents’ experiences with error disclosure. When applicable, questions used a 4-point Likert scale (Strongly Agree to Strongly Disagree). T-tests were used to assess for statistically significant differences between participant responses pre-post workshop. Study statistics were computed using Microsoft Excel.

We also performed a cost analysis for this educational program. We collected the number of hours and cost associated with the efforts described above and reported an overall per participant time and cost estimate to undertake this simulation-based training workshop in medical error disclosure.

## Results

Over the course of the one-year study period, a total of 27 out of a class of 31 (87.0%) pediatric level 2 (PL2) residents completed the workshop. Twenty-three of the participants (85.2%) were female. No additional demographic data were collected on study participants. All study participants completed both follow-up surveys.

One-week follow-up data

All participants felt that the workshop included simulated cases there were relevant to training (agree: n=11, 40.7%; strongly agree: n=16, 59.3%), were relevant to current clinical duties (agree: n=10, 37.0%; strongly agree: n=17, 63.0%), and were realistic (agree: n=11, 40.7%; strongly agree: n=16, 59.3%), and that this workshop was a valuable addition to his/her training (agree: n=18, 66.7%; strongly agree: n=9, 33.3%). The majority of participants felt that this workshop was presented at an appropriate time during training (agree: n=20, 74.1%; strongly agree: n=6, 22.2%) and 33.3% (n=9) of study participants stated that they would like to repeat this workshop prior to completion of their training.

All participants felt that workshop participation increased his/her confidence to recognize that a PSE has occurred (agree: n=13, 48.1%; strongly agree: n=14, 51.9%) and felt that workshop participation improved their ability to carry out the disclosure conversation (agree: n=18, 66.7%; strongly agree: n=9, 33.3%).

After participating in this workshop, residents self-reported changes in decision-making in the event of a PSE (Table 1). Notably, study participants indicated that after receiving the training they were more likely to engage the attending physician, risk management, and patient relations in the disclosure conversation (p <=0.05).

**Table 1:** Resident self-reported changes in engaging other members of the health care team in the disclosure conversation pre and post workshop

	Pre n (%) N=27	Post n (%) N=27	p-value
Attending Physician	15(55.6)	25(92.6)	<0.01
Fellow	24 (88.9)	23(85.2)	0.69
Senior Resident	22(81.5)	18(66.7)	0.22
Another Resident	12(44.4)	9(33.3)	0.41
Patient's Family	22(81.5)	23(85.2)	0.72
Patient	16(59.3)	22(81.5)	0.08
Primary Nurse	17(63.0)	21(77.8)	0.24
Risk Management	2(7.4)	24(88.9)	< 0.01
Patient Relations	2(7.4)	12(44.4)	<0.01
Hospital Event Reporting System	20(74.1)	23(85.2)	0.32

Three-month follow-up data

At three-month follow-up, nine study participants (9/27=33.3%) reported involvement in disclosure of a PSE since workshop participation, with four of these nine study participants reporting personally disclosing to the patient/family. All study participants who reported involvement in disclosure of a PSE felt the workshop adequately prepared them for this experience (agree: n=6, 66.7%; strongly agree: n=3, 33.3%).

Qualitative comments

**Figure 3:**
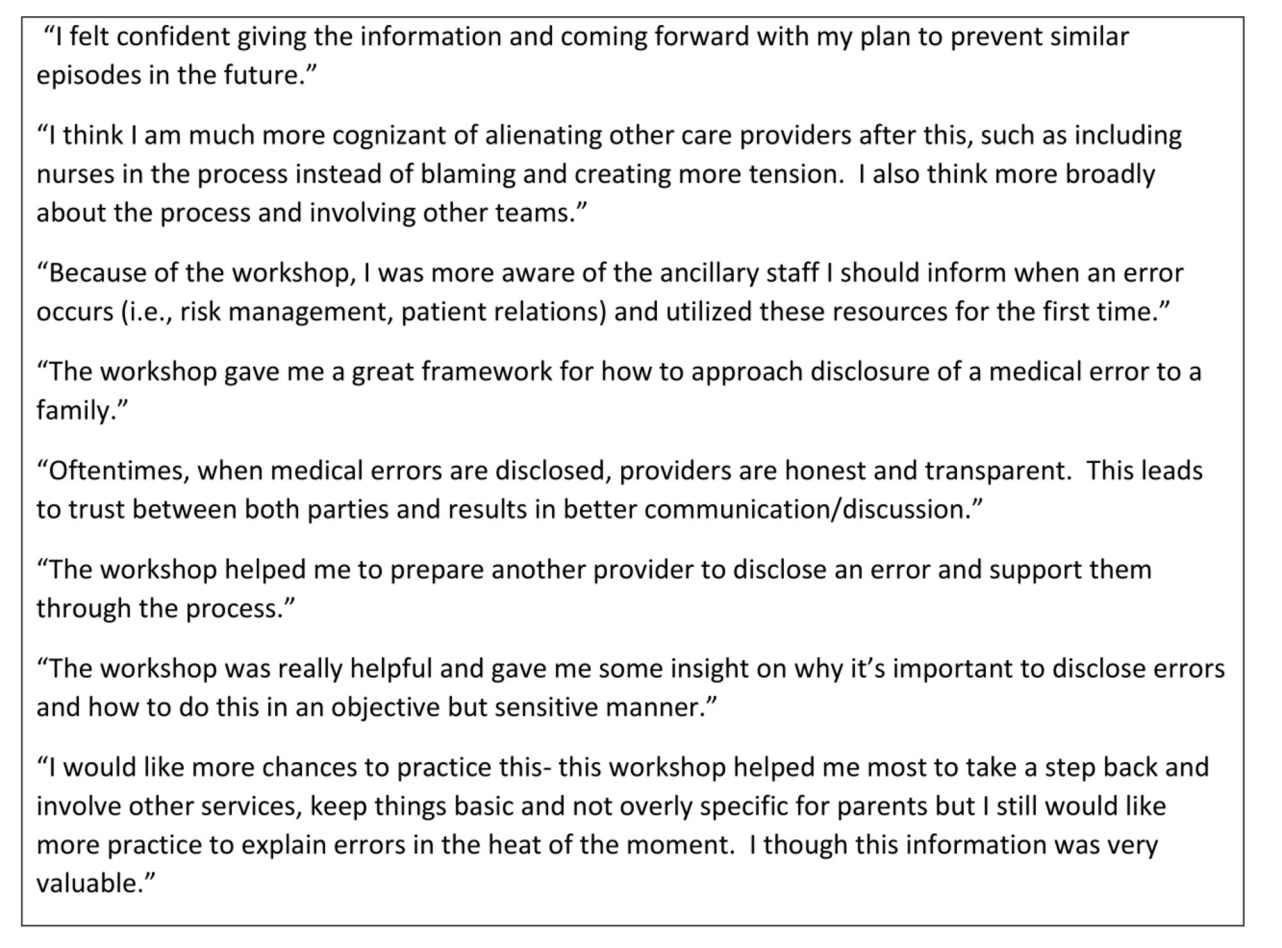
Participant qualitative comments

From the comment sections in both follow-up surveys, a total of 27 comments were made. Common themes in the comments included improved confidence and preparedness with information sharing and decision-making in the setting of a PSE, improved understanding of resources available to clinicians when a PSE occurs, and desire for more practice with difficult conversations. Selected comments are highlighted in Figure 3.

Resource utilization

Our interprofessional team consisted of six core faculty members, comprising both physicians and nurses. All faculty members held active clinical roles at our institution, had prior experience and formal training in simulation education, and had previously participated in a formal simulation debriefing course. In general, cost categories in simulation-based education include equipment and materials, personnel, facility costs, required client (or learner) inputs and resources, and other unaccounted for program inputs, such as technologies, etc. According to a systematic review looking into the costs of simulation-based medical education, a simulated scenario with a high-fidelity simulator costs $259 per resident, when compared to $5 per resident for a traditional lecture [19]. This cost is inclusive of simulation center time and personnel resources. Using this framework, we can estimate that this workshop costs $6,993 for one residency class per year. We must also consider the time-cost for the physician and nurse facilitators, who each spent 39 hours teaching over the course of the year.

## Discussion

This small, single-center study found that all study participants reported increased confidence and preparedness in their ability to lead the initial disclosure conversation after completing the workshop. In addition to reporting that they were more likely to engage their attending physician, risk management, and patient relations in the disclosure conversation, the workshop was shown to be relevant to actual clinical practice given that nine (one-third of) study participants reported involvement in the disclosure of a PSE in the three months after participation. All nine felt that the workshop content helped them with this experience. More notably, four of those nine study participants reported disclosing to the patient/family themselves. While the sample size is small, these data are encouraging and suggest a sustained impact that focused, immersive, and simulation-based educational sessions can have on trainees.

This curriculum addresses an educational need within pediatric training and supports prior literature on barriers to disclosure to the pediatric patient. A prior study on pediatrician attitudes toward disclosure found that most believed that PSE could be disclosed to developmentally appropriate pediatric patients (mean age 12 years) and believe that physicians and parents should jointly decide whether to disclose to pediatric patients [20]. However, they also believe that the training programs in disclosure should include formal instruction on disclosure and offer the opportunity to both practice these skills and receive feedback [7].

The release of the Institute of Medicine (IOM) report “To Err Is Human” was a key driver for the development and implementation of initiatives, policies, programs, and laws to address medical safety [21]. While some organizations have opted to use dedicated disclosure teams, many physicians and professional groups believe that all health care practitioners should receive training, education, and practice with this complex skill. In fact, a recent statement from the American Academy of Pediatrics Committee on Medical Liability and Risk Management states that full disclosure of PSE is an ethical obligation for pediatricians [3]. As the patient’s advocate, the physician plays a key role in the disclosure of a PSE, providing the patient and family with an explanation of what happened and the assurance that steps are being taken to prevent a similar occurrence in the future. A successful training session in error disclosure must stimulate individual reflection, group discussion, and provide a framework for clinicians and educators to approach this challenging discussion. The overarching goal of our workshop is to prepare pediatric residents to handle disclosing a PSE in the immediate setting, to deal with a patient and/or parent’s emotional reaction, to respond to questions regarding how the error occurred, to recognize their own emotions when discussing medical errors, and to provide them with the skills to help them carry out these conversations for the remainder of their residency and in their future careers.

Simulation-based training is uniquely suited for this type of training in that it offers participants an opportunity to push past the boundaries of their comfort, make mistakes in a safe learning environment, and receive corrective feedback for future practice [15]. A simulation-based workshop in disclosure of PSE provides physicians with the opportunity for hands-on practice of this complex skill. One main barrier is the cost of simulation training, which may be prohibitive to the sustainability of an immersive simulation-based training program in the absence of high level institutional commitment and support. However, cost-effectiveness in medical education often cannot be linked to actual outcomes but rather is tied to improved patient safety and intangible long-term societal benefits. Additionally, the disclosure training cost may not reflect its value given that the failure to disclose PSE promptly and properly can disrupt patient-clinician relationships, thereby increasing the chance of malpractice lawsuits [8]. The possibility of even one lawsuit avoided due to good communication may make an investment into a resource-intensive training program worthwhile.

There are a number of limitations to our study. This workshop was implemented at a single center, and participants surveyed may be influenced by local or institutional beliefs and therefore results may not be generalizable to other institutions. In addition, we only enrolled second-year pediatric residents and cannot directly comment how physicians at other levels of training or practice or other health care providers would benefit from such training programs. A principal critique of studies involving an educational intervention is that an improvement in participant confidence is expected and therefore not a noteworthy result; however, in this case we are teaching a psychosocial skill rather than a psychomotor one, and we do believe that improved learner confidence is a more powerful indicator of training success. Additionally, the fact that pediatrics residents participated – and even led – the error disclosure conversation following their participation in our workshop speaks to this. One final limitation is that although our study shows promise as an effective, interactive method for teaching error disclosure to trainees, it is relatively expensive and may not be affordable to all institutions. However, we believe that the traditional lecture format incompletely addresses learner needs in this emotionally and psychologically complex task; we suggest partnering with local experts in medical education to develop a multimodal training curriculum in disclosure, taking into account local resources in order to maximize educational efficacy for your learners.

Our study suggests that while hands-on practice is critical, the faculty and simulation resources needed for implementation of such workshops may not be sustainable long-term. Future training curricula should integrate didactic presentations with ample opportunity for hands-on practice of this critical communication skill. Future work on this subject should explore the long-term retention of skills by participants, impact on clinical practice, and implementation of similar curricula to include other health care providers.

## Conclusions

Health care providers need to be equipped to manage the immediate aftermath of a patient safety event. This small, single-center study found that immersive simulation is uniquely suited for teaching the difficult conversation skills required for disclosure of patient safety events. Hands-on practice is vital to becoming facile with this complex skill. Through our innovative workshop, providers gain practical knowledge, experience, and confidence in disclosure. In addition, the workshop was shown to be helpful in actual clinical practice, which may offset its cost. The sustainability of a comprehensive immersive training curriculum in disclosure ultimately requires institutional commitment to support clinicians in best practices related to patient safety and communication.
